# Life satisfaction as compared with traditional risk factors in relation to incident cardiovascular diseases

**DOI:** 10.1007/s10654-025-01225-w

**Published:** 2025-04-07

**Authors:** Minghao Kou, Xiang Li, Hao Ma, Xuan Wang, Yoriko Heianza, JoAnn E. Manson, Lu Qi

**Affiliations:** 1 Department of Epidemiology, Celia Scott Weatherhead School of Public Health and Tropical Medicine, Tulane University, 1440 Canal Street, Suite 1724, New Orleans, LA 70112 USA; 2 Division of Endocrinology, Diabetes and Metabolism, Department of Medicine, College of Medicine, University of Illinois Chicago, Chicago, IL USA; 3 Department of Nutrition, Harvard T.H. Chan School of Public Health, Boston, MA USA; 4 Department of Medicine, Brigham and Women’s Hospital, Harvard Medical School, Boston, MA USA; 5 Department of Epidemiology, Harvard T.H. Chan School of Public Health, Boston, MA USA

**Keywords:** Life satisfaction, Well-being, Cardiovascular disease

## Abstract

**Background:**

Emerging evidence suggests a role of psychological well-being in the development of cardiovascular disease (CVD), but supportive data remain limited. This study assessed the prospective associations between life satisfaction and incident CVD, as well as the relative importance of life satisfaction compared to traditional risk factors.

**Methods:**

The study included 153,810 participants free of CVD at baseline, with measurements of life satisfaction on general happiness, personal health, family relationships, friendships, and financial situation, followed up until December 31, 2022. Cox proportional hazards models were used to estimate associations between life satisfaction and incident CVD. The relative importance of life satisfaction in predicting CVD was measured by explained R^2^ values.

**Results:**

During a median follow-up of 12.9 years, 14,370 incident CVD events occurred, including 10,070 CHD and 2,895 strokes. Individuals with low life satisfaction had an 80% higher risk of CVD compared to those with high life satisfaction (hazard ratio [95% confidence interval], 1.84 [1.63–2.07] for CVD, 1.83 [1.59–2.10] for CHD, and 1.74 [1.31–2.31] for stroke). Life satisfaction was ranked as the fourth-strongest CVD risk factor, following hypertension, race, and income. Low satisfaction with all individual aspects was significantly associated with higher risks of CVD and CHD (*P* < 0.05), while satisfaction with personal health showing the strongest association.

**Conclusions:**

This study indicates that life satisfaction is robustly associated with incident CVD and may be considered one of the strongest predictors of CVD risk, alongside traditional risk factors. Our findings support the inclusion of life satisfaction in cardiovascular health metrics.

**Supplementary Information:**

The online version contains supplementary material available at 10.1007/s10654-025-01225-w.

## Introduction

Cardiovascular disease (CVD) is the leading cause of death worldwide, taking 17.9 million lives each year [1]. A group of health behaviors (smoking, physical activity, diet, and weight control) and biochemical factors (cholesterol, blood pressure, and glucose control) have been consistently related to CVD risk. The American Heart Association proposed metrics for Life’s Essential 8 [2], including these factors along with sleep duration, in the assessment of cardiovascular health (CVH). Despite growing recognition of the fundamental role of psychosocial factors, such as the mind-heart-body connection [3], in the development of CVD, current CVH metrics focus predominantly on behavioral and biochemical risk factors. The omission of psychosocial factors is largely due to the paucity of supportive evidence exploring their associations with CVD.

Well-being is an important psychosocial factor that captures how individuals experience and evaluate various aspects of their lives. Well-being can be broadly categorized into two domains: objective well-being, which encompasses factors like socioeconomic status and environmental conditions, and subjective well-being, which reflects personal assessments of life satisfaction, happiness, and affective states [4, 5]. Among subjective measures, life satisfaction is commonly used to assess overall well-being. Previous studies have shown that subjective well-being, assessed through life satisfaction, is associated with cardiovascular risk factors (behavioral and biological) and atherosclerosis [6–11]. In addition, well-being is a multifaceted construct, and examining individual indicators of life satisfaction will enable us to explore which specific aspects of well-being are most strongly related to CVD risk. We hypothesized that different domains might contribute differently to CVD risk, given the multifaceted nature of well-being and its potential pathways to health outcomes. Only one study to date has associated both the broad scope and specific domains of life satisfaction with coronary heart disease (CHD) risk [12]. This study, however, was limited by its generalizability, its method of classification, and a relatively small sample size.

Therefore, the current study investigates the prospective associations between life satisfaction, assessed across five domains (general happiness, family relationships, friendships, health, and financial situation), and incident CVD in adults from the UK Biobank study. We also compare the predictive value of life satisfaction relative to traditional CVD risk factors, calculating its relative importance in predicting incident CVD.

## Methods

### Study population

The UK Biobank study is a population-based cohort study which recruited more than 500,000 participants from the general population at 22 assessment centers throughout the United Kingdom, aged 40–69 years when recruited at baseline between 2006 and 2010. A total of 172,654 participants who underwent the touchscreen questionnaire on life satisfaction (general happiness, and satisfaction with personal health, family relationships, friendships, and financial situation) were included. We finally included 153,810 participants, after excluding 6,540 participants with incomplete information on life satisfaction components, and 12,206 participants with prevalent CVD and heart failure at baseline (Supplemental Fig. 1). The study was approved by the Northwest Multi-Centre Research Ethics Committee, and written informed consent was obtained from all participants.

### Assessment of life satisfaction

The overall life satisfaction in our study was assessed based on five key components: general happiness, satisfaction with family, friendship, personal health, and financial situation. These items were collected at baseline using a 6-point reverse-scored Likert scale (1 = extremely happy, and 6 = extremely unhappy), with responses to the following questions: “In general, how happy are you?” and “In general, how satisfied are you with your family relationships/friendships/health/financial situation?”. The responses were summed up to create an overall life satisfaction score ranging from 5 to 30. Given the inherently categorical nature of the response format, we chose to classify life satisfaction into three levels: high (scores 5–10, corresponding to an average of “very happy”), medium (scores 11–19, corresponding to a range from “very happy” to “moderately unhappy”), and low (scores 20–30, corresponding to an average of “moderately unhappy”). A single composite of domain was used in previous studies [12, 13], and showed good stability [14]. 

Categorizing continuous data may result in the loss of meaningful variance. However, our decision was informed by the fact that the Likert scale used to assess life satisfaction was inherently categorical, with predefined levels of response. Simply treating life satisfaction as a continuous variable could introduce artificial precision that does not align with the original measurement scale. To balance these concerns, we included both continuous and categorical life satisfaction in our analysis.

### Assessment of outcomes

Participants’ hospital inpatient records were linked to obtain data on admissions, diagnoses, and deaths. Information on death and death date was obtained from death certificates held by the *National Health Service*. Incident outcomes (CVD, CHD, and stroke) were identified using the following *International Statistical Classification of Diseases and Related Health Problems*,* Tenth Revision (ICD-10)* codes: CVD (I20-25, I63, I64, and G45), CHD (I21-25), and stroke (I60, I61, and I63-64) [15]. Previous studies have shown that using medical records to identify incident CVD provided reliable and accurate outcomes [16, 17]. Follow-up time was calculated from the date of baseline to the diagnosis date of the first cardiovascular event, the date of death, or the censoring date (31 Dec 2022), whichever occurred first. Detailed information on the ascertainment of outcomes is available online at https://biobank.ctsu.ox.ac.uk/showcase/label.cgi?id=2000.

### Assessment of covariates

The covariates included age, sex, race, education levels, Townsend deprivation index, income, body mass index (BMI), smoking status, alcohol consumption, healthy diet, physical activity, baseline hypertension, diabetes, high cholesterol, loneliness and social isolation. Detailed information about assessment of covariates was described in the Supplemental Material (Supplemental Methods and Supplemental Table 1).

### Statistical analysis

Missing data were imputed with multiple imputation by the fully conditional specification method [18, 19]. We imputed five complete datasets and all variables (overall life satisfaction, incident CVD, follow-up time, and covariates) were included in the imputation model.

Baseline characteristics and follow-up time of the study population were summarized by life satisfaction levels in the originally unimputed dataset. The hazard ratio (HR) and 95% confidence interval (CI) were estimated to evaluate the association between life satisfaction and each outcome using Cox proportional hazards models. The Kaplan-Meier method and Schoenfeld residuals were used to evaluate the proportional hazards assumption, and no violation was found. Models were adjusted for age, sex, race (White, or non-White), education levels (low, medium, or high), Townsend deprivation index (< median, or ≥ median), BMI, smoking status (never, or ever smoker), alcohol consumption (moderate, or non-moderate), healthy diet (yes, or no), physically active (yes, or no), baseline hypertension (yes, or no), diabetes (yes, or no), and high cholesterol (yes, or no). We also plotted the cumulative hazard of CVD by life satisfaction levels across the whole follow-up period and displayed the P value for the log-rank test. The associations between life satisfaction components and incident CVD were also evaluated with the same covariates, where “very unhappy” or “extremely unhappy” responses were grouped together due to the small proportion of study population. Pooled results across all five imputed datasets were finally reported.

For overall life satisfaction, we analyzed its relative importance, which provided an estimate of the importance of specific factors in terms of predicting outcomes, calculated with R function coxphERR in package *clinfun* [20]. We measured the relative importance of all variables in the abovementioned model by calculating R^2^ values of models with sex as a stratum and age as the time scale [21]. We finally reported the relative importance (explained R^2^ values) across five imputed datasets.

Subgroup analyses were performed to evaluate the consistency of the association between overall life satisfaction and CVD across different populations in the first imputed dataset as a representation. Multiplicative interactions were assessed by modeling the same covariates and the interaction terms for each outcome. The interaction was tested using the Wald test.

We performed sensitivity analyses to test the robustness of the study. First, we reported the results with complete-case analysis (*n* = 109,916) although imputed datasets should be identical with complete cases, theoretically. Second, to minimize the probability of reverse causation, we excluded those participants who had incident CVD within the first two years of follow-up. Third, we further adjusted for social isolation and loneliness to test if the associations were independent of other psychosocial factors.

All analyses were performed using SAS version 9.4 (SAS Institute Inc. Cary, NC, USA) and RStudio version 4.2.2. A two-tailed P value of < 0.05 was considered statistically significant.

## Results

### Study population

The baseline characteristics of the study population were shown in Table 1. 23.6%, 74.9%, and 1.5% of 153,810 participants, respectively, had high, medium, and low life satisfaction. Participants in the group with low life satisfaction were more likely to be men, non-Whites, ever smokers, moderate alcohol users, and physically inactive. They were also more likely to have lower socioeconomic status, higher BMI, baseline diabetes, baseline high cholesterol, and lower healthy diet frequency. In addition, participants with low overall life satisfaction were younger, and less likely to have baseline hypertension.

**Table 1:** Characteristics of the study population by overall life satisfaction

	Life satisfaction levels		
	High (*n* = 36,303)	Medium (*n* = 115,133)	Low (*n* = 2,374)
Age, years	57.6 (8.0)	56.1 (8.2)	51.8 (7.3)
Men, %	43.7	44.1	49
White, %	94.4	91.2	79.4
Education level, %			
Low	33.1	31.7	35.8
Medium	17.9	17.8	15.9
High	49.1	50.6	48.3
Townsend deprivation index	-1.7 (2.7)	-1.1 (2.9)	0.8 (3.4)
Income, %			
Less than £18‚000	15.7	21.8	49
£18‚000 to £30‚999	24.3	25.7	20.5
£31‚000 to £51‚999	26.5	26.6	18
£52‚000 to £100‚000	23.9	20.7	10.7
Greater than £100‚000	9.6	5.2	1.8
Body mass index, kg/m²	26.7 (4.3)	27.4 (4.8)	29.5 (6.4)
Never smoking, %	59.5	55.6	46.8
Moderate alcohol consumption, %	66.7	68.4	73
Physical active, %	68	59.4	43.3
Healthy diet, %	41.3	36.3	28.8
Hypertension, %	53.9	52.8	50.7
Diabetes, %	3.3	5.1	10.7
High cholesterol, %	14.4	15.7	19.5
Follow-up time, years	12.9 (12.6–13.2)	12.9 (12.6–13.2)	12.8 (12.5–13.2)

Values are means (SD) or median (IQR) for continuous variables; percentages for categorical variables

### The association between life satisfaction and incident cardiovascular events

**Fig. 1:**
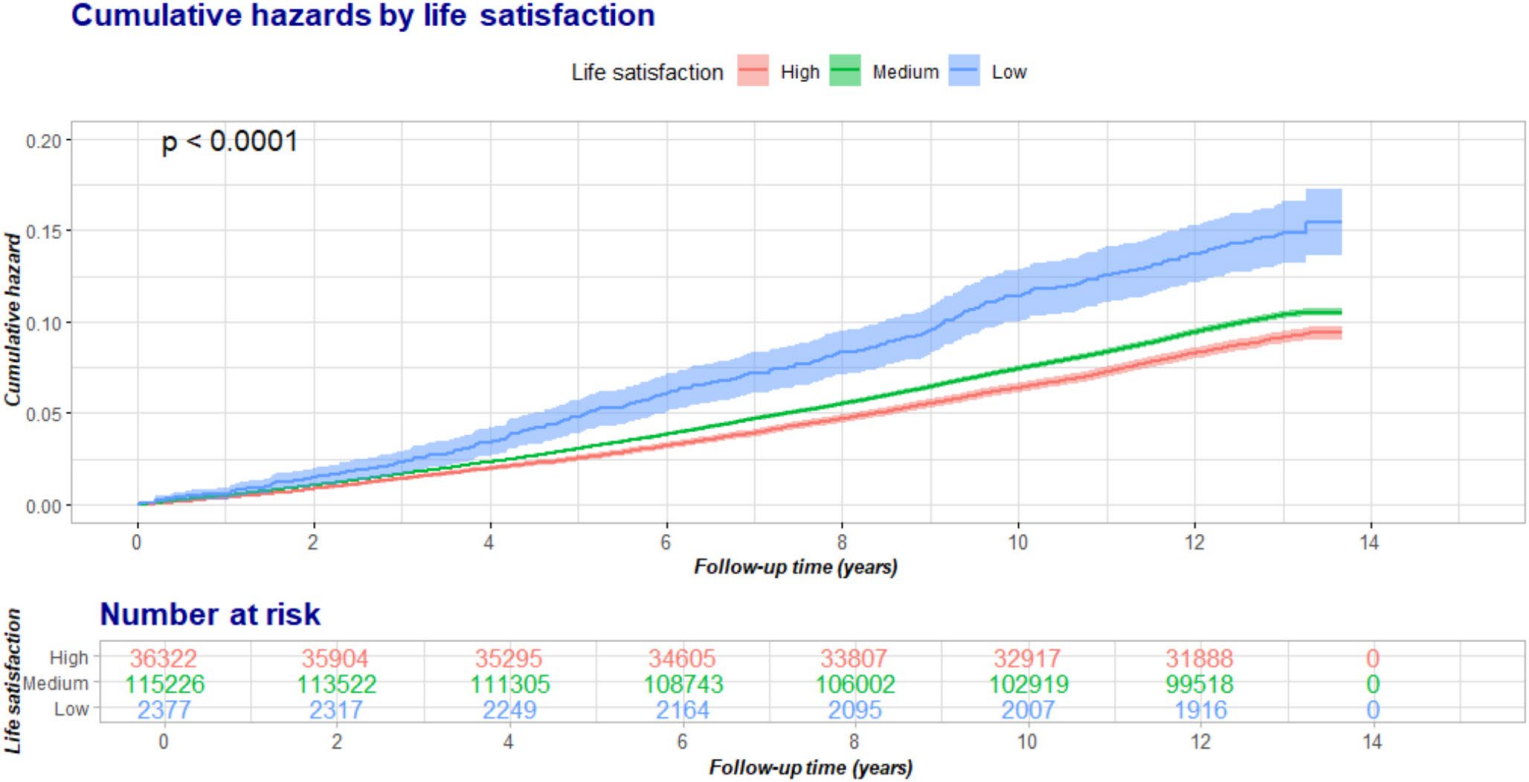
The cumulative hazard of incident cardiovascular disease by overall life satisfaction

During a median follow-up of 12.9 years, a total of 14,370 incident CVD events were identified, including 10,070 incident CHD, 2,895 incident stroke. The cumulative hazards were significantly distinct across life satisfaction levels, and the group with low life satisfaction had the highest cumulative hazard of CVD (Fig. 1). Table 2 shows the adjusted HRs and 95% CI for each outcome, according to life satisfaction. We observed consistently higher risks of cardiovascular events associated with lower life satisfaction. Compared to the group with high life satisfaction, low life satisfaction was associated with an 84% (HR [95%CI], 1.84 [1.63–2.07]), 83% (1.84 [1.59–2.10]), and 74% (1.74 [1.31–2.31]) higher risk of CVD, CHD, and stroke, respectively. The HR for per-unit increment in life satisfaction was 1.05 (1.04–1.05), 1.05 (1.04–1.06), and 1.05 (1.03–1.06) for CVD, CHD, and stroke. The associations with incident cardiovascular events were different across individual aspects of life satisfaction (Table 3). Satisfaction with personal health was the domain showing the strongest association with incident cardiovascular events, starting from participants with moderate happy attitudes. Satisfaction with family relationships and financial status were also associated with incident outcomes from participants feeling moderate happy with smaller HRs. Satisfaction with friendship was the domain with the weakest associations with outcomes, that only participants feeling very/extremely unhappy were associated with significantly higher risks of CVD and CHD.

**Table 2:** The adjusted hazard ratio of cardiovascular events by life satisfaction^a^

Outcomes	Life satisfaction levels			Life satisfaction scores, per-unit increment
	High	Medium	Low	
CVD	ref	1.18 (1.13–1.22)	1.84 (1.63–2.07)	1.05 (1.04–1.05)
CHD	ref	1.17 (1.11–1.23)	1.83 (1.59–2.10)	1.05 (1.04–1.06)
Stroke	ref	1.16 (1.06–1.27)	1.74 (1.31–2.31)	1.05 (1.03–1.06)

Abbreviation: CVD, cardiovascular disease; CHD, coronary heart disease

a. Model adjusted for age, sex, White race, education level, Townsend deprivation index, income, body mass index, smoking status, moderate alcohol consumption, physical activity, healthy diet, hypertension, diabetes, and high cholesterol

**Table 3:** The adjusted hazard ratio of cardiovascular events by aspects of life satisfaction^a^

Outcomes	Levels	Aspects of life satisfaction				
		General Happiness	Health	Family	Friendship	Financial
CVD	Extremely happy	ref	ref	ref	ref	ref
	Very happy	0.96 (0.89–1.03)	1.04 (0.95–1.14)	1.01 (0.97–1.06)	1.01 (0.96–1.06)	1.00 (0.94–1.07)
	Moderately happy	1.07 (0.99–1.14)	1.39 (1.27–1.52)	1.07 (1.02–1.12)	1.04 (0.99–1.10)	1.09 (1.02–1.17)
	Moderately unhappy	1.24 (1.11–1.38)	1.80 (1.63–1.99)	1.16 (1.07–1.27)	1.09 (0.97–1.22)	1.28 (1.18–1.40)
	Very or extremely unhappy	1.61 (1.36–1.90)	2.39 (2.13–2.67)	1.43 (1.27–1.60)	1.54 (1.29–1.85)	1.46 (1.32–1.60)
CHD	Extremely happy	ref	ref	ref	ref	ref
	Very happy	1.00 (0.92–1.09)	1.00 (0.89–1.11)	1.00 (0.95–1.05)	0.99 (0.93–1.05)	1.01 (0.93–1.09)
	Moderately happy	1.10 (1.01–1.20)	1.36 (1.22–1.51)	1.06 (1.01–1.12)	1.03 (0.96–1.09)	1.10 (1.02–1.20)
	Moderately unhappy	1.30 (1.14–1.48)	1.74 (1.55–1.95)	1.15 (1.04–1.28)	1.05 (0.91–1.20)	1.31 (1.18–1.44)
	Very or extremely unhappy	1.76 (1.45–2.13)	2.30 (2.01–2.63)	1.40 (1.22–1.60)	1.61 (1.31–1.99)	1.47 (1.31–1.64)
Stroke	Extremely happy	ref	ref	ref	ref	ref
	Very happy	0.85 (0.73–1.01)	0.97 (0.80–1.17)	1.06 (0.96–1.17)	1.03 (0.92–1.15)	1.02 (0.87–1.19)
	Moderately happy	1.01 (0.87–1.18)	1.24 (1.03–1.49)	1.13 (1.02–1.26)	1.09 (0.96–1.23)	1.09 (0.94–1.27)
	Moderately unhappy	1.05 (0.82–1.35)	1.49 (1.21–1.85)	1.23 (1.01–1.49)	1.08 (0.82–1.41)	1.38 (1.14–1.66)
	Very or extremely unhappy	1.52 (1.04–2.21)	1.94 (1.52–2.49)	1.76 (1.39–2.25)	0.95 (0.56–1.62)	1.50 (1.20–1.86)

Abbreviation: CVD, cardiovascular disease; CHD, coronary heart disease

a. Model adjusted for age, sex, White race, education level, Townsend deprivation index, income, body mass index, smoking status, moderate alcohol consumption, physical activity, healthy diet, hypertension, diabetes, and high cholesterol

The results were robustly consistent in sensitivity analyses. With the complete-case analysis, we found no apparent differences for CVD and CHD (Supplemental Table 2). The HR for incident stroke comparing low vs. high life satisfaction changed from 1.74 (1.31–2.31) to 1.93 (1.36–2.74), probably due to the relatively low incidence. For individual components, slightly changed HRs did not alter the associations (Supplemental Table 3). To minimize the probability of reverse causation bias, we excluded incident CVD events within the first two years of follow-up and found slight changes, which would not influence the interpretations of the associations (Supplemental Tables 4 and 5). Supplemental Tables 6 and 7 showed that further adjustment for psychosocial factors, isolation and loneliness, did not change the associations significantly, demonstrating the value of life satisfaction independent of other psychosocial factors.

Subgroup analyses showed that the associations between overall life satisfaction and CVD might be modified by age, sex, and baseline hypertension. Women had significantly higher HRs of CVD as compared to men (HR [95%CI], low vs. high life satisfaction, 2.32 [1.94–2.76] vs. 1.58 [1.35–1.86], P for interaction = 0.005, Supplemental Table 8). Stronger associations were also observed for older participants (≥ 60 years old) and those with baseline hypertension (P for interaction < 0.05). For incident CHD, the associations with overall life satisfaction were also stronger among older participants and women (P for interaction < 0.05, Supplemental Table 9). The interactions were not significant for stroke, possibly due to the relatively low incidence of stroke (Supplemental Table 10).

### Relative importance of life satisfaction

**Fig. 2:**
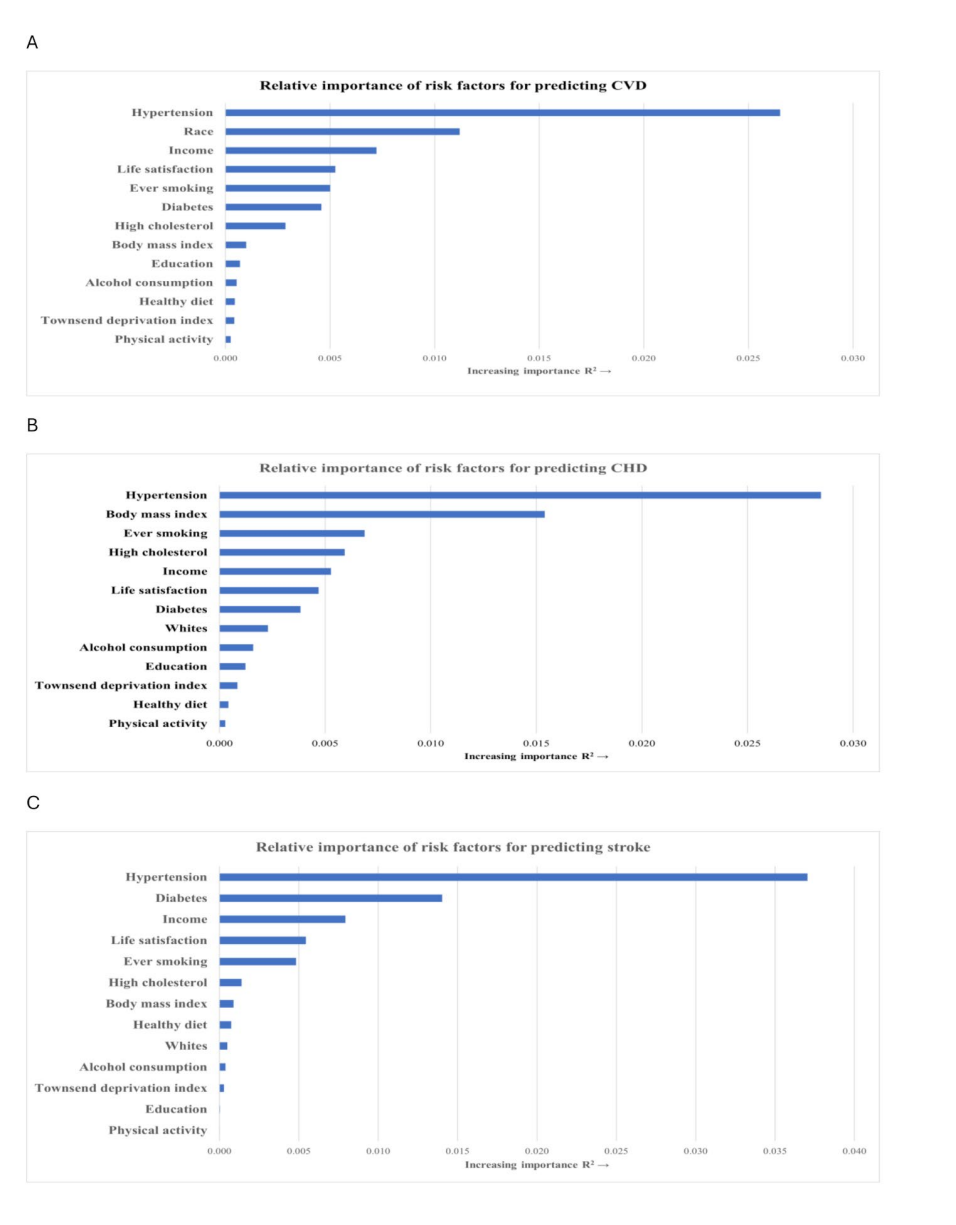
Relative importance of variables for predicting cardiovascular events. (**A**) cardiovascular disease; (**B**) coronary heart disease; and (**C**) ischemic stroke. Cox proportional hazards model adjusted for Whites race, education level, Townsend deprivation index, income, body mass index, smoking status, moderate alcohol consumption, physical activity, healthy diet, hypertension, diabetes, and high cholesterol

We then compared the relative importance of life satisfaction with traditional risk factors for cardiovascular events (Supplemental Table 11). Life satisfaction was ranked as the 4th strongest predictor for incident CVD, following hypertension, race, and income (Fig. 2A). As for incident CHD, life satisfaction was the 6th strongest predictor (Fig. 2B), following hypertension, BMI, smoking, high cholesterol, and income. The relative importance of life satisfaction was also high for incident stroke as the 4th strongest predictor, following hypertension, diabetes and income (Fig. 2C).

## Discussion

In this prospective cohort study with a median follow-up of 12.9 years, we found that low life satisfaction was associated with an 80% higher risk of CVD compared to those with high life satisfaction. Lack of satisfaction with personal health, family, and financial situation was consistently related to a higher CVD risk, with satisfaction with personal health showing the strongest association. Based on the relative importance ranking, life satisfaction was the fourth strongest risk factor for CVD, following well-established risk factors such as hypertension, race and income.

The American Heart Association highlighted the potential importance of psychological well-being and mind-heart-body connection in the primordial prevention of CVD [3]. In our study, we examined both a composite measure of life satisfaction and individual indicators to better understand their roles in predicting incident CVD. The composite score provided a broad overview of life satisfaction, while the analysis of individual domains (e.g., general happiness and personal health) highlighted aspects of well-being that were more strongly associated with CVD. These findings suggest that well-being should be viewed as a multifaceted construct, where both overall life satisfaction and specific components contribute to cardiovascular health in distinct ways. Notably, life satisfaction regarding personal health showed the strongest association with incident CVD, likely because it reflects an individual’s overall health condition. Achieving high well-being may indeed help prevent CVD, and practical strategies include fostering strong social connections [22, 23], engaging in regular physical activity [24], practicing mindfulness and stress management [25], and expressing gratitude [11]. These approaches can promote better emotional regulation and healthier lifestyle choices, which collectively contribute to improved well-being and CVH.

Our findings align with previous research. For example, in a church-based intervention, low life satisfaction is consistently correlated with more adverse weight-related factors (waist, abdomen and hip circumferences and BMI) [7], and poorer eating habits [6]. Longitudinal studies have also suggested a prospective association of high subjective well-being with lower glycated hemoglobin [10], and better health behaviors (combining dietary habits, physical activity, alcohol consumption, and smoking status) [9]. Boehm JK et al. found a significantly lower risk of incident CHD associated with higher overall life satisfaction, as well as several domains including job, family life, sex life, and self-satisfaction [12]. Our measure of life satisfaction, which captures multiple domains, provides a broad and comprehensive assessment of individual well-being. However, it is subject to potential biases, including social desirability and variability in individual perceptions. Furthermore, while this measure has been widely used in large cohort studies, its use in clinical practice requires further validation. Future studies should explore whether these questions, or more concise versions of them, could be integrated into clinical settings to provide valuable insights into patient health and well-being.

To our knowledge, this is the first study to assess the relative importance of life satisfaction in comparison to other well-established risk factors for predicting CVD. Interestingly, our analysis ranked life satisfaction as one of the most important risk factors, underscoring its influential role in CVD development. These findings might have substantial implications in disease prevention. Current CVH metrics (e.g., Life’s Essential 8) acknowledge the relevance of psychosocial factors but do not specifically include them. Our results suggest that incorporating life satisfaction into CVH metrics may add valuable information for assessing and predicting CVD risk. Moreover, given the different patterns of the relative importance and associations of life satisfaction with CHD and stroke, it is possible that achieving high life satisfaction may prevent certain CVD subtypes better than others, calling for additional research. Cautious interpretations are necessary, given that the variability of relative importance could be affected by selection of variables, model specifications, and study population. Although the R² values in this analysis are relatively small, this is consistent with findings from other studies investigating the relative importance of risk factors for CVD and other health outcomes [26, 27]. Small R² values are commonly observed for individual risk factors, when multiple factors contribute to complex outcomes like CVD. Despite their small magnitude, the R² values provide valuable information about the relative contribution of each factor to the prediction of CVD risk.

The underlying mechanisms through which low life satisfaction is linked to higher risk of CVD are likely multifactorial. Life satisfaction may be associated with a higher risk of CVD with CVH as mediators. However, the bidirectional association between life satisfaction and CVH, including blood pressure [28], BMI [29], and physical activity [30] suggests shared risk factors for CVD [2]. Nevertheless, the associations between life satisfaction and CVD were significant even after rigorous adjustment for these CVH metrics, indicating an independent relationship. In addition, life satisfaction might promote protective effects of other psychosocial factors (e.g., optimism) and mitigate the harmful influence of negative factors, thus further influencing the risk of CVD [31]. Importantly, our results did not change after adjusting for loneliness and social isolation, emphasizing a unique link between life satisfaction and CVD. Given the observational design of this study, no causal inference should be made with identified associations, while further research is needed to investigate potential causality.

The major strength of this study included a prospective cohort, a large sample size and long follow-up durations, allowing us to assess the associations between life satisfaction, its components, and specific subtypes of CVD with sufficient power. Another notable strength of this study is the consistency of risk estimates and relative importance across cardiovascular outcomes, which reinforces the robustness and generalizability of our findings across different cardiovascular endpoints. The study’s limitations also warrant consideration. First, we used multiple imputation to avoid potential loss of power, and the results might be subject to imputation errors. However, the results were consistent in multiple sensitivity analyses, suggesting the robustness of the findings. Second, the generalizability of the study may be limited due to potential “healthy volunteer” selection bias and the predominantly White racial composition of the sample. Additionally, the findings may not be fully generalizable to low- and middle-income countries, where socioeconomic and cultural factors may influence well-being evaluations [32, 33]. Third, the use of self-reported measures introduces the potential for measurement errors, including biases such as social desirability, which could affect life satisfaction reporting. Future studies should explore the most reliable methods for incorporating life satisfaction measures into clinical practice. Fourth, residual confounding may exist despite extensive adjustment for covariates, as any indicators of a favorable life (e.g., income, neighborhood characteristics) could influence both life satisfaction and CVD risks.

In conclusion, our study demonstrates robust associations between life satisfaction — both overall and within its individual components — and the risk of incident CVD. These associations are comparable in magnitude to those of several well-established CVD risk factors, such as hypertension and smoking. Our findings suggest that life satisfaction should be considered as an important factor in CVH metrics for evaluating CVH and the risk of incident CVD.

## Electronic supplementary material

Below is the link to the electronic supplementary material.

Supplementary Material 1

Supplementary Material 2

## Abbreviations

BMI: Body mass index
CHD: Coronary heart disease
CVD: Cardiovascular disease
CVH: Cardiovascular health
HR: Hazard ratio
ICD-10: International Classification of Diseases, Tenth Revision

## Declarations

All authors have reported that they have no relationships relevant to the contents of this paper to disclose.
