# Supplementary material for: Life satisfaction as compared with traditional risk factors in relation to incident cardiovascular diseases
Source: Eur J Epidemiol. 2025 Apr 7;40(4):441–9. doi: 10.1007/s10654-025-01225-w (PMC12145285; doi:10.1007/s10654-025-01225-w)
Supplement: Supplementary file 1 — Supplementary Material 1 [file 10654_2025_1225_MOESM1_ESM.docx]

**Life satisfaction as compared with traditional risk factors in relation to incident cardiovascular diseases**

Minghao Kou, MHS,^1^ Xiang Li, MD, PhD,^1,2^ Hao Ma, MD, PhD,^1^ Xuan Wang, MD, PhD,^1^ Yoriko Heianza, PhD, RD,^1,3^ JoAnn E. Manson, MD, DrPH,^4,5^ Lu Qi, MD, PhD^1,3^

1. Department of Epidemiology, Celia Scott Weatherhead School of Public Health and Tropical Medicine, Tulane University, New Orleans, LA;

2. Division of Endocrinology, Diabetes and Metabolism, Department of Medicine, College of Medicine, University of Illinois Chicago, Chicago, IL;

3. Department of Nutrition, Harvard T.H. Chan School of Public Health, Boston, MA

4. Department of Medicine, Brigham and Women’s Hospital, Harvard Medical School, Boston, MA;

5. Department of Epidemiology, Harvard T.H. Chan School of Public Health, Boston, MA

**Supplemental Methods**

**Assessment of covariates**

A touch-screen questionnaire was used to assess most of the covariates at baseline.^1^ Sex was defined as men or women. Self-reported race was classified as White or non-White. The highest qualification achieved was converted to years of education and categorized into low (≤10 years), medium education (11-18 years), and high education (≥19 years).^2^ Townsend deprivation index is a composite measure of deprivation based on unemployment, non-car ownership, non-home ownership, and household overcrowding; a negative value represents high socioeconomic status. Body mass index (BMI) was calculated as weight in kilograms divided by height in meters squared (kg/m²). Self-reported smoking status was classified into two categories: never smokers, and ever smokers. Alcohol intake was grouped into two categories: moderate consumption (defined as 0 to 14 g/d for women and 0 to 28 g/d for men, with the maximum limit reflecting US dietary guidelines),^3,4^ and non-moderate consumption (Supplemental Table 1). Physical activity was defined per the Physical Activity Guidelines for Americans as active (>150 minutes of moderate intensity activity per week or >75 minutes of vigorous activity per week or an equivalent combination per week), or inactive.^5^ Healthy diet was based on consumption of at least 4 of 7 food groups (fruits, vegetables, fish, processed meat, unprocessed red meat, whole grains, and refined grains) following recommendations on dietary priorities for cardiometabolic health (Supplemental Table 1).^6^ Blood pressure was double measured using an electronic blood pressure monitor (Omron 705 IT, OMRON Healthcare Europe B.V., Hoofddorp, Netherlands), or using a mercury sphygmomanometer, by a trained nurse at the assessment center. Baseline hypertension was defined as a systolic blood pressure ≥ 140 mmHg or a diastolic blood pressure ≥ 90 mm Hg, a self-reported diagnosis by physicians, blood pressure medication use, or based on the onset time of hypertension (based on ICD-9/10 codes). Baseline diabetes was ascertained based on the onset time of type 1 diabetes, type 2 diabetes, and other types of diabetes (Source: ICD-9 250; ICD-10 E10, E11, E12, E13, E14), self-reported diagnosis by physicians, excluding women with gestational diabetes only. Baseline high cholesterol was ascertained based on a self-reported diagnosis by physicians or lipid-lowering drug use. The loneliness was assessed through two questions, “Do you often feel lonely?” and “How often are you able to confide in someone close to you?” The social isolation scale was measured with three questions: (i) “Including yourself, how many people are living together in your household?”; (ii) “How often do you visit friends or family or have them visit you?”; (iii) “Which of the following leisure/social activities do you engage in once a week or more often?”. The scale of loneliness or social isolation was calculated by summing the individual scores of the two or three corresponding factors, respectively.^7^

**Supplemental Figure**

**
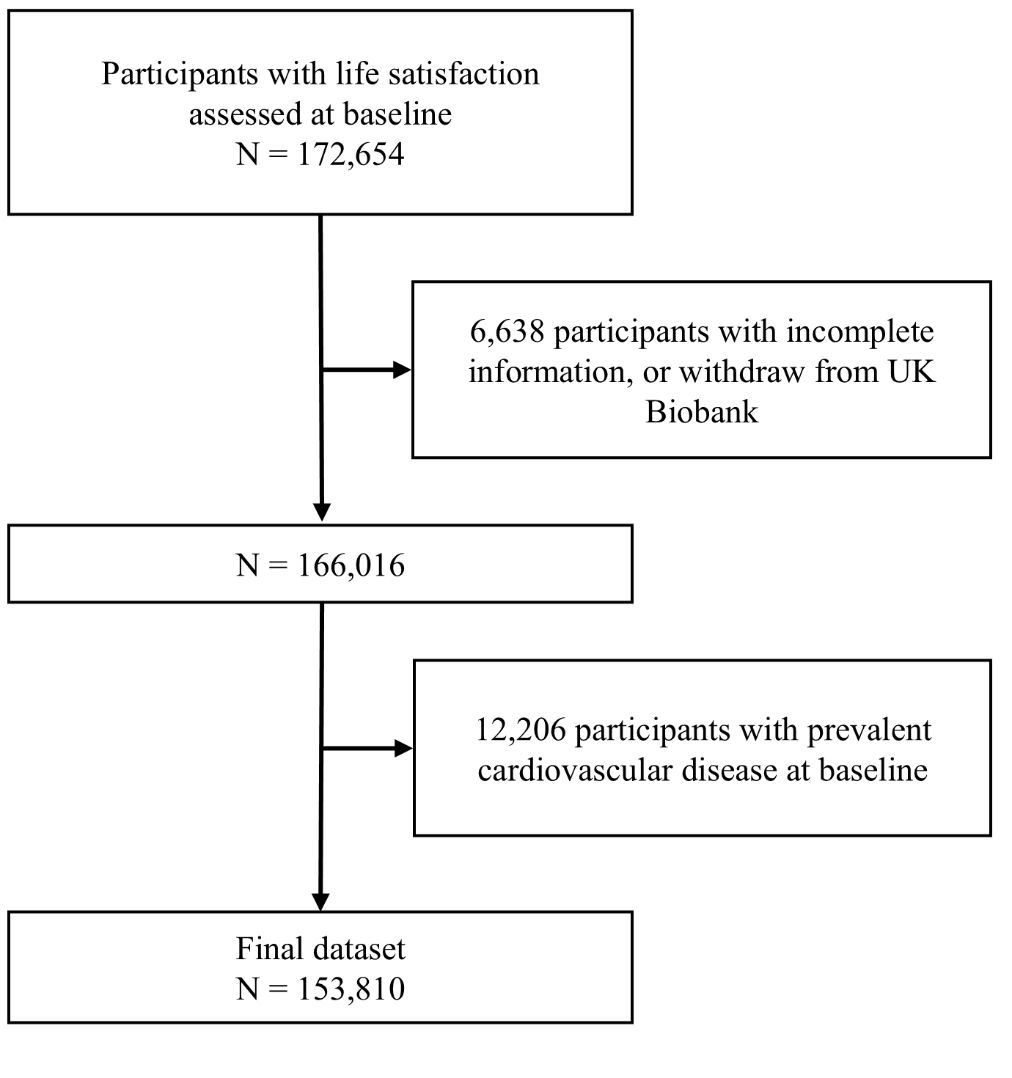
**

Supplemental Figure 1. Cohort flow

**Supplemental Tables**

| **Supplemental Table 1. Definition of lifestyle factors** | | |
| --- | --- | --- |
| **Lifestyle factors** | **Categories** | **Values assigned** |
| Smoking status |  |  |
|  | - Never smoker | 1 |
|  | - Previous smoker | 0 |
|  | - Current smoker | 0 |
| Alcohol consumption | Drink-equivalents were calculated per guidelines, by multiplying the volume in ounces by the alcohol content in percent and dividing by 0.6 ounces of alcohol per drink-equivalent: 125ml wine=0.85 drink-equivalents, 4% ABV pint beer = 1.28 drink-equivalents, 25ml spirits=0.57 drink-equivalents, 50ml fortified wine= 0.56 drink-equivalents. Then drink-equivalents were converted to grams:  1 drink-equivalent = 14g of pure alcohol. |  |
|  | - For men, 0 to 28 g/d | 1 |
|  | - For women, 0 to 14 g/d | 1 |
|  | - Others | 0 |
| Physical activity |  |  |
|  | - ≥150 minutes of moderate intensity activity or ≥75 minutes of vigorous activity or an equivalent combination per week | 1 |
|  | - <150 minutes of moderate intensity activity and <75 minutes of vigorous activity or an equivalent combination per week | 0 |
| Healthy diet | Healthy diet was based on consumption of at least 4 of 7 food groups |  |
|  | - Fruits: ≥ 3 servings/day | 1 |
|  | - Vegetables: ≥ 3 servings/day | 1 |
|  | - Fish: ≥2 servings/week | 1 |
|  | - Processed meats: ≤ 1 serving/week | 1 |
|  | - Unprocessed red meats: ≤ 1.5 servings/week | 1 |
|  | - Whole grains: ≥ 3servings/day | 1 |
|  | - Refined grains: ≤1.5servings/day | 1 |
|  | - Others | 0 |

| **Supplemental Table 2. The complete case analysis of adjusted hazard ratio of cardiovascular events by life satisfaction^a^** | | | | |
| --- | --- | --- | --- | --- |
| Outcomes | Life satisfaction levels | | | Life satisfaction scores, per-unit increment |
|  | High | Medium | Low |  |
| CVD | ref | 1.17 (1.12-1.23) | 1.88 (1.62-2.18) | 1.05 (1.04-1.05) |
| CHD | ref | 1.17 (1.10-1.24) | 1.86 (1.57-2.22) | 1.05 (1.04-1.06) |
| Stroke | ref | 1.20 (1.08-1.34) | 1.93 (1.36-2.74) | 1.05 (1.03-1.07) |
| Abbreviation: CVD, cardiovascular disease; CHD, coronary heart disease | | | | |
| a. Model adjusted for age, sex, White race, education level, Townsend deprivation index, income, body mass index, smoking status, moderate alcohol consumption, physical activity, healthy diet, hypertension, diabetes, and high cholesterol | | | | |

| **Supplemental Table 3. The complete case analysis of adjusted hazard ratio of cardiovascular events by aspects of life satisfaction^a^** | | | | | | |
| --- | --- | --- | --- | --- | --- | --- |
| Outcomes | Levels | Aspects of life satisfaction | | | | |
|  |  | General Happiness | Health | Family | Friendship | Financial |
| CVD | Extremely happy | ref | ref | ref | ref | ref |
|  | Very happy | 1.00 (0.92-1.09) | 1.05 (0.94-1.17) | 1.00 (0.95-1.05) | 0.99 (0.93-1.05) | 0.96 (0.88-1.04) |
|  | Moderately happy | 1.11 (1.01-1.21) | 1.38 (1.24-1.53) | 1.06 (1.01-1.12) | 1.02 (0.95-1.09) | 1.07 (0.99-1.16) |
|  | Moderately unhappy | 1.24 (1.08-1.41) | 1.84 (1.64-2.07) | 1.17 (1.05-1.29) | 1.03 (0.90-1.19) | 1.25 (1.13-1.38) |
|  | Very or extremely unhappy | 1.75 (1.42-2.15) | 2.35 (2.05-2.70) | 1.39 (1.20-1.60) | 1.47 (1.16-1.85) | 1.43 (1.27-1.60) |
| CHD | Extremely happy | ref | ref | ref | ref | ref |
|  | Very happy | 1.04 (0.94-1.16) | 1.00 (0.88-1.14) | 0.98 (0.92-1.04) | 0.98 (0.91-1.06) | 0.96 (0.87-1.06) |
|  | Moderately happy | 1.14 (1.02-1.27) | 1.36 (1.20-1.55) | 1.03 (0.97-1.11) | 1.01 (0.93-1.09) | 1.09 (0.99-1.19) |
|  | Moderately unhappy | 1.30 (1.11-1.52) | 1.79 (1.56-2.06) | 1.14 (1.01-1.29) | 1.00 (0.85-1.18) | 1.27 (1.13-1.43) |
|  | Very or extremely unhappy | 1.96 (1.54-2.48) | 2.23 (1.89-2.63) | 1.39 (1.17-1.64) | 1.54 (1.19-2.01) | 1.43 (1.25-1.64) |
| Stroke | Extremely happy | ref | ref | ref | ref | ref |
|  | Very happy | 0.85 (0.70-1.03) | 0.97 (0.77-1.22) | 1.09 (0.96-1.23) | 0.99 (0.86-1.14) | 0.94 (0.79-1.13) |
|  | Moderately happy | 1.04 (0.86-1.25) | 1.21 (0.97-1.51) | 1.19 (1.04-1.36) | 1.06 (0.92-1.23) | 1.07 (0.89-1.27) |
|  | Moderately unhappy | 1.03 (0.76-1.40) | 1.49 (1.15-1.92) | 1.35 (1.08-1.70) | 1.03 (0.74-1.42) | 1.36 (1.09-1.70) |
|  | Very or extremely unhappy | 1.66 (1.04-2.65) | 1.88 (1.38-2.56) | 1.60 (1.16-2.20) | 0.93 (0.48-1.82) | 1.38 (1.06-1.81) |
| Abbreviation: CVD, cardiovascular disease; CHD, coronary heart disease | | | | | | |
| a. Model adjusted for age, sex, White race, education level, Townsend deprivation index, income, body mass index, smoking status, moderate alcohol consumption, physical activity, healthy diet, hypertension, diabetes, and high cholesterol | | | | | | |

| **Supplemental Table 4. The adjusted hazard ratio of cardiovascular events by life satisfaction after excluding first two-year follow-up^a^** | | | | |
| --- | --- | --- | --- | --- |
| Outcomes | Life satisfaction levels | | | Life satisfaction scores, per-unit increment |
|  | High | Medium | Low |  |
| CVD | ref | 1.16 (1.12-1.21) | 1.82 (1.61-2.07) | 1.05 (1.04-1.05) |
| CHD | ref | 1.15 (1.09-1.21) | 1.79 (1.54-2.08) | 1.05 (1.04-1.05) |
| Stroke | ref | 1.17 (1.07-1.29) | 1.91 (1.43-2.56) | 1.05 (1.03-1.06) |
| Abbreviation: CVD, cardiovascular disease; CHD, coronary heart disease | | | | |
| a. Model adjusted for age, sex, White race, education level, Townsend deprivation index, income, body mass index, smoking status, moderate alcohol consumption, physical activity, healthy diet, hypertension, diabetes, and high cholesterol | | | | |

| **Supplemental Table 5. The adjusted hazard ratio of cardiovascular events by aspects of life satisfaction after excluding first two-year follow-up^a^** | | | | | | |
| --- | --- | --- | --- | --- | --- | --- |
| Outcomes | Levels | Aspects of life satisfaction | | | | |
|  |  | General Happiness | Health | Family | Friendship | Financial |
| CVD | Extremely happy | ref | ref | ref | ref | ref |
|  | Very happy | 0.94 (0.87-1.01) | 1.04 (0.94-1.14) | 1.02 (0.98-1.07) | 1.02 (0.97-1.08) | 1.02 (0.95-1.10) |
|  | Moderately happy | 1.04 (0.96-1.12) | 1.36 (1.24-1.49) | 1.08 (1.03-1.14) | 1.05 (0.99-1.11) | 1.11 (1.03-1.19) |
|  | Moderately unhappy | 1.21 (1.08-1.36) | 1.71 (1.54-1.90) | 1.16 (1.06-1.27) | 1.11 (0.98-1.25) | 1.30 (1.19-1.43) |
|  | Very or extremely unhappy | 1.52 (1.27-1.81) | 2.29 (2.03-2.58) | 1.44 (1.28-1.63) | 1.53 (1.26-1.86) | 1.47 (1.33-1.63) |
| CHD | Extremely happy | ref | ref | ref | ref | ref |
|  | Very happy | 0.96 (0.88-1.05) | 0.99 (0.89-1.11) | 1.01 (0.96-1.07) | 1.00 (0.94-1.07) | 1.02 (0.94-1.12) |
|  | Moderately happy | 1.05 (0.96-1.15) | 1.32 (1.19-1.48) | 1.07 (1.01-1.14) | 1.03 (0.96-1.10) | 1.11 (1.02-1.21) |
|  | Moderately unhappy | 1.24 (1.08-1.42) | 1.65 (1.46-1.87) | 1.14 (1.02-1.27) | 1.05 (0.91-1.21) | 1.30 (1.17-1.45) |
|  | Very or extremely unhappy | 1.61 (1.31-1.98) | 2.22 (1.93-2.56) | 1.40 (1.22-1.62) | 1.58 (1.26-1.97) | 1.47 (1.31-1.66) |
| Stroke | Extremely happy | ref | ref | ref | ref | ref |
|  | Very happy | 0.87 (0.74-1.03) | 0.94 (0.77-1.14) | 1.09 (0.98-1.21) | 1.06 (0.94-1.20) | 1.03 (0.88-1.21) |
|  | Moderately happy | 1.02 (0.87-1.20) | 1.23 (1.01-1.49) | 1.16 (1.03-1.3) | 1.12 (0.98-1.28) | 1.09 (0.93-1.28) |
|  | Moderately unhappy | 1.04 (0.80-1.36) | 1.41 (1.13-1.76) | 1.23 (1.01-1.52) | 1.15 (0.87-1.53) | 1.39 (1.14-1.70) |
|  | Very or extremely unhappy | 1.64 (1.11-2.42) | 1.83 (1.41-2.38) | 1.88 (1.46-2.41) | 1.11 (0.65-1.89) | 1.55 (1.24-1.95) |
| Abbreviation: CVD, cardiovascular disease; CHD, coronary heart disease | | | | | | |
| a. Model adjusted for age, sex, White race, education level, Townsend deprivation index, income, body mass index, smoking status, moderate alcohol consumption, physical activity, healthy diet, hypertension, diabetes, and high cholesterol | | | | | | |

| **Supplemental Table 6. The adjusted hazard ratio of cardiovascular events by life satisfaction further adjusting for loneliness and social isolation^a^** | | | | |
| --- | --- | --- | --- | --- |
| Outcomes | Life satisfaction levels | | | Life satisfaction scores, per-unit increment |
|  | High | Medium | Low |  |
| CVD | ref | 1.16 (1.12-1.21) | 1.78 (1.57-2.02) | 1.05 (1.04-1.05) |
| CHD | ref | 1.16 (1.11-1.22) | 1.76 (1.51-2.05) | 1.05 (1.04-1.06) |
| Stroke | ref | 1.13 (1.03-1.24) | 1.66 (1.22-2.25) | 1.04 (1.03-1.06) |
| Abbreviation: CVD, cardiovascular disease; CHD, coronary heart disease | | | | |
| a. Model adjusted for age, sex, White race, education level, Townsend deprivation index, income, body mass index, smoking status, moderate alcohol consumption, physical activity, healthy diet, hypertension, diabetes, high cholesterol, loneliness, social isolation | | | | |

| **Supplemental Table 7. The adjusted hazard ratio of cardiovascular events by aspects of life satisfaction further adjusting for loneliness and social isolation^a^** | | | | | | |
| --- | --- | --- | --- | --- | --- | --- |
| Outcomes | Levels | Aspects of life satisfaction | | | | |
|  |  | General Happiness | Health | Family | Friendship | Financial |
| CVD | Extremely happy | ref | ref | ref | ref | ref |
|  | Very happy | 0.98 (0.91-1.05) | 1.05 (0.96-1.16) | 1.01 (0.97-1.06) | 1.00 (0.95-1.05) | 0.99 (0.92-1.06) |
|  | Moderately happy | 1.06 (0.98-1.14) | 1.40 (1.27-1.53) | 1.06 (1.01-1.11) | 1.02 (0.96-1.08) | 1.08 (1.01-1.16) |
|  | Moderately unhappy | 1.19 (1.06-1.34) | 1.83 (1.65-2.02) | 1.14 (1.04-1.24) | 1.06 (0.94-1.20) | 1.25 (1.14-1.36) |
|  | Very or extremely unhappy | 1.53 (1.28-1.83) | 2.42 (2.15-2.72) | 1.35 (1.20-1.53) | 1.43 (1.18-1.74) | 1.40 (1.27-1.55) |
| CHD | Extremely happy | ref | ref | ref | ref | ref |
|  | Very happy | 1.02 (0.93-1.11) | 1.02 (0.91-1.14) | 1.00 (0.95-1.06) | 0.98 (0.92-1.04) | 1.00 (0.92-1.09) |
|  | Moderately happy | 1.10 (1.01-1.20) | 1.37 (1.23-1.53) | 1.05 (0.99-1.12) | 1.00 (0.94-1.07) | 1.10 (1.01-1.20) |
|  | Moderately unhappy | 1.25 (1.09-1.44) | 1.78 (1.58-2.01) | 1.13 (1.02-1.26) | 1.02 (0.88-1.18) | 1.30 (1.18-1.45) |
|  | Very or extremely unhappy | 1.66 (1.36-2.04) | 2.30 (2.00-2.65) | 1.31 (1.14-1.52) | 1.49 (1.19-1.86) | 1.43 (1.27-1.61) |
| Stroke | Extremely happy | ref | ref | ref | ref | ref |
|  | Very happy | 0.85 (0.73-1.01) | 0.98 (0.80-1.19) | 1.07 (0.96-1.18) | 1.01 (0.90-1.14) | 0.98 (0.84-1.15) |
|  | Moderately happy | 0.99 (0.85-1.16) | 1.24 (1.03-1.51) | 1.11 (0.99-1.24) | 1.04 (0.91-1.18) | 1.05 (0.90-1.22) |
|  | Moderately unhappy | 1.02 (0.79-1.33) | 1.48 (1.19-1.85) | 1.21 (0.98-1.48) | 1.05 (0.79-1.39) | 1.25 (1.03-1.52) |
|  | Very or extremely unhappy | 1.48 (0.99-2.20) | 1.97 (1.52-2.55) | 1.68 (1.30-2.18) | 0.73 (0.40-1.35) | 1.38 (1.10-1.73) |
| Abbreviation: CVD, cardiovascular disease; CHD, coronary heart disease | | | | | | |
| a. Model adjusted for age, sex, White race, education level, Townsend deprivation index, income, body mass index, smoking status, moderate alcohol consumption, physical activity, healthy diet, hypertension, diabetes, and high cholesterol | | | | | | |

| **Supplemental Table 8. Subgroup analyses for association between life satisfaction and incident CVD** | | | | |
| --- | --- | --- | --- | --- |
|  | Life satisfaction levels | | | P for interaction |
|  | High | Medium | Low |  |
| Age |  |  |  | 0.028 |
| <60 years old | ref | 1.24 (1.17-1.31) | 1.84 (1.58-2.13) |  |
| ≥60 years old | ref | 1.15 (1.10-1.20) | 1.99 (1.64-2.41) |  |
| Sex |  |  |  | 0.005 |
| Women | ref | 1.22 (1.14-1.30) | 2.32 (1.94-2.76) |  |
| Men | ref | 1.15 (1.09-1.21) | 1.58 (1.35-1.86) |  |
| Race |  |  |  | 0.804 |
| Non-Whites | ref | 1.23 (1.04-1.45) | 2.01 (1.52-2.66) |  |
| Whites | ref | 1.17 (1.13-1.22) | 1.82 (1.59-2.08) |  |
| Education level |  |  |  | 0.242 |
| Low | ref | 1.24 (1.17-1.33) | 1.85 (1.53-2.23) |  |
| Medium | ref | 1.14 (1.04-1.26) | 1.70 (1.26-2.29) |  |
| High | ref | 1.13 (1.07-1.21) | 1.91 (1.60-2.27) |  |
| Townsend deprivation index |  |  |  | 0.992 |
| <median | ref | 1.18 (1.11-1.24) | 1.86 (1.49-2.33) |  |
| ≥median | ref | 1.18 (1.11-1.25) | 1.85 (1.60-2.13) |  |
| Smoking |  |  |  | 0.434 |
| Ever | ref | 1.17 (1.11-1.24) | 1.96 (1.68-2.28) |  |
| Never | ref | 1.18 (1.12-1.25) | 1.70 (1.41-2.05) |  |
| Alcohol consumption |  |  |  | 0.726 |
| High | ref | 1.16 (1.09-1.25) | 1.95 (1.57-2.44) |  |
| Moderate | ref | 1.18 (1.13-1.24) | 1.81 (1.58-2.09) |  |
| Physical active |  |  |  | 0.808 |
| Inactive | ref | 1.20 (1.11-1.29) | 1.91 (1.61-2.27) |  |
| Active | ref | 1.17 (1.11-1.23) | 1.81 (1.53-2.13) |  |
| Healthy diet |  |  |  | 0.047 |
| No | ref | 1.16 (1.11-1.21) | 1.78 (1.57-2.02) |  |
| Yes | ref | 1.32 (1.18-1.46) | 2.36 (1.72-3.23) |  |
| Baseline hypertension |  |  |  | 0.043 |
| No | ref | 1.09 (1.01-1.18) | 1.63 (1.32-2.02) |  |
| Yes | ref | 1.21 (1.16-1.27) | 1.95 (1.69-2.25) |  |
| Baseline diabetes |  |  |  | 0.949 |
| No | ref | 1.18 (1.13-1.23) | 1.86 (1.63-2.13) |  |
| Yes | ref | 1.19 (1.03-1.37) | 1.80 (1.35-2.39) |  |
| Baseline high cholesterol |  |  |  | 0.686 |
| No | ref | 1.17 (1.11-1.22) | 1.83 (1.58-2.11) |  |
| Yes | ref | 1.21 (1.12-1.31) | 1.91 (1.55-2.35) |  |
| Model adjusted for age, sex, White race, education level, Townsend deprivation index, income, body mass index, smoking status, moderate alcohol consumption, physical activity, healthy diet, hypertension, diabetes, and high cholesterol | | | | |

| **Supplemental Table 9. Subgroup analyses for association between life satisfaction and incident CHD** | | | | |
| --- | --- | --- | --- | --- |
|  | Life satisfaction levels | | | P for interaction |
|  | High | Medium | Low |  |
| Age |  |  |  | 0.023 |
| <60 years old | ref | 1.25 (1.17-1.34) | 1.89 (1.59-2.24) |  |
| ≥60 years old | ref | 1.14 (1.08-1.20) | 1.90 (1.52-2.38) |  |
| Sex |  |  |  | 0.009 |
| Women | ref | 1.24 (1.14-1.35) | 2.39 (1.93-2.97) |  |
| Men | ref | 1.14 (1.07-1.21) | 1.58 (1.32-1.89) |  |
| Race |  |  |  | 0.717 |
| Non-Whites | ref | 1.12 (0.93-1.35) | 1.91 (1.39-2.63) |  |
| Whites | ref | 1.18 (1.12-1.24) | 1.80 (1.54-2.11) |  |
| Education level |  |  |  | 0.237 |
| Low | ref | 1.24 (1.15-1.34) | 1.88 (1.51-2.34) |  |
| Medium | ref | 1.14 (1.01-1.27) | 1.50 (1.03-2.18) |  |
| High | ref | 1.12 (1.05-1.21) | 1.93 (1.58-2.36) |  |
| Townsend deprivation index |  |  |  | 0.698 |
| <median | ref | 1.19 (1.12-1.28) | 1.86 (1.42-2.43) |  |
| ≥median | ref | 1.15 (1.07-1.23) | 1.81 (1.54-2.14) |  |
| Smoking |  |  |  | 0.866 |
| Ever | ref | 1.16 (1.08-1.24) | 1.85 (1.55-2.22) |  |
| Never | ref | 1.19 (1.11-1.27) | 1.82 (1.47-2.25) |  |
| Alcohol consumption |  |  |  | 0.321 |
| High | ref | 1.14 (1.05-1.24) | 2.08 (1.61-2.67) |  |
| Moderate | ref | 1.19 (1.12-1.26) | 1.76 (1.49-2.08) |  |
| Physical active |  |  |  | 0.576 |
| Inactive | ref | 1.13 (1.03-1.23) | 1.74 (1.42-2.13) |  |
| Active | ref | 1.19 (1.12-1.26) | 1.91 (1.58-2.31) |  |
| Healthy diet |  |  |  | 0.087 |
| No | ref | 1.15 (1.09-1.21) | 1.76 (1.51-2.04) |  |
| Yes | ref | 1.30 (1.14-1.48) | 2.51 (1.75-3.60) |  |
| Baseline hypertension |  |  |  | 0.108 |
| No | ref | 1.08 (0.99-1.18) | 1.83 (1.43-2.33) |  |
| Yes | ref | 1.21 (1.14-1.28) | 1.84 (1.55-2.18) |  |
| Baseline diabetes |  |  |  | 0.583 |
| No | ref | 1.17 (1.11-1.23) | 1.91 (1.64-2.22) |  |
| Yes | ref | 1.18 (1.01-1.39) | 1.61 (1.15-2.24) |  |
| Baseline high cholesterol |  |  |  | 0.441 |
| No | ref | 1.15 (1.09-1.22) | 1.83 (1.54-2.17) |  |
| Yes | ref | 1.23 (1.12-1.35) | 1.90 (1.50-2.40) |  |
| Model adjusted for age, sex, White race, education level, Townsend deprivation index, income, body mass index, smoking status, moderate alcohol consumption, physical activity, healthy diet, hypertension, diabetes, and high cholesterol | | | | |

| **Supplemental Table 10. Subgroup analyses for association between life satisfaction and incident stroke** | | | | |
| --- | --- | --- | --- | --- |
|  | Life satisfaction levels | | | P for interaction |
|  | High | Medium | Low |  |
| Age |  |  |  | 0.982 |
| <60 years old | ref | 1.18 (1.03-1.34) | 1.75 (1.22-2.52) |  |
| ≥60 years old | ref | 1.16 (1.05-1.28) | 1.74 (1.12-2.70) |  |
| Sex |  |  |  | 0.511 |
| Women | ref | 1.16 (1.02-1.32) | 2.07 (1.38-3.11) |  |
| Men | ref | 1.17 (1.04-1.32) | 1.52 (1.03-2.24) |  |
| Race |  |  |  | 0.692 |
| Non-Whites | ref | 1.36 (0.91-2.04) | 1.79 (0.87-3.7) |  |
| Whites | ref | 1.15 (1.05-1.26) | 1.77 (1.30-2.42) |  |
| Education level |  |  |  | 0.916 |
| Low | ref | 1.21 (1.05-1.39) | 1.73 (1.10-2.71) |  |
| Medium | ref | 1.09 (0.89-1.34) | 1.47 (0.72-3.01) |  |
| High | ref | 1.16 (1.01-1.32) | 1.87 (1.23-2.82) |  |
| Townsend deprivation index |  |  |  | 0.190 |
| <median | ref | 1.08 (0.96-1.22) | 1.76 (1.05-2.96) |  |
| ≥median | ref | 1.28 (1.11-1.46) | 1.84 (1.30-2.58) |  |
| Smoking |  |  |  | 0.467 |
| Ever | ref | 1.22 (1.07-1.39) | 1.95 (1.36-2.79) |  |
| Never | ref | 1.11 (0.98-1.26) | 1.50 (0.95-2.37) |  |
| Alcohol consumption |  |  |  | 0.326 |
| High | ref | 1.23 (1.06-1.43) | 1.33 (0.72-2.44) |  |
| Moderate | ref | 1.13 (1.01-1.26) | 1.88 (1.37-2.59) |  |
| Physical active |  |  |  | 0.023 |
| Inactive | ref | 1.42 (1.19-1.70) | 2.36 (1.58-3.53) |  |
| Active | ref | 1.09 (0.98-1.20) | 1.44 (0.96-2.16) |  |
| Healthy diet |  |  |  | 0.620 |
| No | ref | 1.14 (1.04-1.26) | 1.73 (1.28-2.34) |  |
| Yes | ref | 1.29 (1.03-1.63) | 1.78 (0.78-4.07) |  |
| Baseline hypertension |  |  |  | 0.653 |
| No | ref | 1.21 (1.02-1.44) | 1.52 (0.87-2.62) |  |
| Yes | ref | 1.15 (1.04-1.27) | 1.84 (1.33-2.56) |  |
| Baseline diabetes |  |  |  | 0.085 |
| No | ref | 1.14 (1.04-1.25) | 1.56 (1.12-2.17) |  |
| Yes | ref | 1.59 (1.13-2.23) | 3.04 (1.67-5.53) |  |
| Baseline high cholesterol |  |  |  | 0.423 |
| No | ref | 1.14 (1.03-1.26) | 1.56 (1.10-2.22) |  |
| Yes | ref | 1.25 (1.03-1.52) | 2.23 (1.39-3.59) |  |
| Model adjusted for age, sex, White race, education level, Townsend deprivation index, income, body mass index, smoking status, moderate alcohol consumption, physical activity, healthy diet, hypertension, diabetes, and high cholesterol | | | | |

| Supplemental Table 11. Relative importance of variables in Cox proportional hazard models | | | |
| --- | --- | --- | --- |
| Variables | Outcomes | | |
|  | CVD | CHD | Stroke |
| Life satisfaction | 0.00525 | 0.00469 | 0.00544 |
| Whites | 0.01119 | 0.00229 | 0.00049 |
| Body mass index | 0.00099 | 0.01540 | 0.00089 |
| Education | 0.00069 | 0.00123 | 0.00004 |
| Income | 0.00721 | 0.00527 | 0.00793 |
| Townsend deprivation index | 0.00042 | 0.00085 | 0.00028 |
| Ever smoking | 0.00500 | 0.00687 | 0.00483 |
| Alcohol consumption | 0.00053 | 0.00160 | 0.00039 |
| Physical activity | 0.00025 | 0.00027 | 0.00002 |
| Healthy diet | 0.00045 | 0.00042 | 0.00074 |
| Hypertension | 0.02651 | 0.02848 | 0.03704 |
| Diabetes | 0.00458 | 0.00383 | 0.01402 |
| High cholesterol | 0.00287 | 0.00593 | 0.00140 |
| Total | 0.11468 | 0.13489 | 0.09329 |
| Abbreviation: CVD, cardiovascular disease; CHD, coronary heart disease | | | |
| Model adjusted for White race, education level, Townsend deprivation index, income, body mass index, smoking status, moderate alcohol consumption, physical activity, healthy diet, hypertension, diabetes, and high cholesterol | | | |

**Reference**

1. Sudlow C, Gallacher J, Allen N, et al. UK Biobank: An Open Access Resource for Identifying the Causes of a Wide Range of Complex Diseases of Middle and Old Age. *PLoS medicine*. 2015;12(3):e1001779. doi:10.1371/journal.pmed.1001779

2. Carter AR, Gill D, Davies NM, et al. Understanding the consequences of education inequality on cardiovascular disease: mendelian randomisation study. *BMJ*. 2019;365:l1855. doi:10.1136/bmj.l1855

3. Lourida I, Hannon E, Littlejohns TJ, et al. Association of Lifestyle and Genetic Risk With Incidence of Dementia. *Jama*. Aug 6 2019;322(5):430-437. doi:10.1001/jama.2019.9879

4. Services UDoHaH. 2015-2020 Dietary guidelines for Americans. Accessed 01/03, 2024. <https://health.gov/sites/default/files/2019-09/2015-2020_Dietary_Guidelines.pdf>

5. Piercy KL, Troiano RP, Ballard RM, et al. The Physical Activity Guidelines for Americans. *Jama*. Nov 20 2018;320(19):2020-2028. doi:10.1001/jama.2018.14854

6. Mozaffarian D. Dietary and Policy Priorities for Cardiovascular Disease, Diabetes, and Obesity: A Comprehensive Review. *Circulation*. Jan 12 2016;133(2):187-225. doi:10.1161/circulationaha.115.018585

7. Elovainio M, Hakulinen C, Pulkki-Råback L, et al. Contribution of risk factors to excess mortality in isolated and lonely individuals: an analysis of data from the UK Biobank cohort study. *The Lancet Public Health*. 2017;2(6):e260-e266. doi:10.1016/S2468-2667(17)30075-0
