# Supplementary material for: Life satisfaction as compared with traditional risk factors in relation to incident cardiovascular diseases
Source: Eur J Epidemiol. 2025 Apr 7;40(4):441–9. doi: 10.1007/s10654-025-01225-w (PMC12145285; doi:10.1007/s10654-025-01225-w)
Supplement: Supplementary file 2 — Supplementary Material 2 [file 10654_2025_1225_MOESM2_ESM.docx]

To. Dr. Albert Hofman

Editor-in-Chief,

*European Journal of Epidemiology*

Dear Dr. Albert Hofman,

Subject: Submission of revised paper Ref. EJEP-D-23-01063R1 - Life satisfaction as compared with traditional risk factors in relation to incident cardiovascular diseases.

We are grateful for your kind email on 03/17/2025 enclosing the reviewers’ valuable comments. We carefully reviewed and addressed the comments point-by-point and revised the manuscript accordingly, which was tracked and colored as red in the manuscript.

Thanks to the reviewers’ comments, we believe our manuscript has been strengthened considerably and hope now it is suitable for publication in the *European Journal of Epidemiology*.

Sincerely,

Minghao Kou, MHS

Lu Qi, MD, PhD, on behalf of coauthors

Department of Epidemiology, School of Public Health and Tropical Medicine, Tulane University

1440 Canal Street, Suite 1724, New Orleans, LA 70112, USA

Telephone: 504-988-3549; Fax: 504-988-1568

Email: lqi1@tulane.edu; [luqi@hsph.harvard.edu](mailto:luqi@hsph.harvard.edu)

**Reviewer comments to the Authors:

Reviewer #1 (Comments to the Authors):**

*General Comments: The authors have substantially and comprenesivel revised the manuscript and addressed all comments and questions stated in the reviews. In particular, they have now provided a detailed explanation of the relative importance measures (R-squared values).*

*However, the following minor issues should be revised before publication:*

**Response**: We greatly appreciate this Reviewer’s positive comments. Please see below our point-by-point responses to individual comments.

**Specific comments:**

*1. In the methods section, the authors mention that "Cox proportional hazards models were performed to evaluate the association between life satisfaction and each outcome."*

*Here, it should be added that hazard ratios with 95% confidence intervals were used to assess the association between predictors and outcomes.*

**Response:** Thank you for your suggestion. We have modified the Method section as follows:

**Statistical Analysis, Page 7-8)** “The hazard ratio (HR) and 95% confidence interval (CI) were estimated to evaluate the association between life satisfaction and each outcome using Cox proportional hazards models.”

*2. It could be added in the discussion section as a strength of the study that the risk estimates are consistent across the three cardiovascular outcomes CVD, CHD and stroke (see Tables 2 and 3)*

**Response:** Thank you for this important suggestion. We believe that the consistency of risk estimates and relative importance is a notable strength because it could support the robustness and generalizability of our findings across different cardiovascular endpoints.

We have added the following sentence to the Discussion section:

**Discussion, Page 15)** “Another notable strength of this study is the consistency of risk estimates and relative importance across cardiovascular outcomes, which reinforces the robustness and generalizability of our findings across different cardiovascular endpoints.”

*3. The study conclusions are well-written and do not overstate the findings. As this is a prediction study, it should be briefly mentioned in the discussion section that this study design does not allow for the assessment of causal inferences.*

**Response:** Thank you for your suggestion. We agree that no causal inference should be made in such an observational study. The current findings could only suggest life satisfaction as a potentially applicable psychological factor in assessing CVD risks. We have added the following sentence in the Discussion section:

**Discussion, Page 14)** “Given the observational design of this study, no causal inference should be made with identified associations, while further research is needed to investigate potential causality.”

*4. There is a typo in the second sentence of the last paragraph: "and the risk of incident CVD. hese ..."*

**Response:** Thank you for this careful comment. We have reviewed the manuscript again to correct similar typos.

**Discussion, Page 15)** “These associations are comparable in magnitude to those of several well-established CVD risk factors, such as hypertension and smoking.”

*5. There is a typo in Figure 2A and 2B and 2C in the title of the horizontal axis: "inceasing".*

**Response:** Thank you for this careful comment. We have corrected these typos in figures.

**Reviewer #2 (Comments to the Authors):**

*General Comments: The authors have responded thoughtfully to all comments and questions. I appreciate their detailed responses to each item, particularly the explanation of the relative importance analysis. The revised manuscript is now strengthened and suitable for publication.*

**Response**: We greatly appreciate this Reviewer’s comments. We appreciate the Reviewer’s valuable comments and suggestions.
